# Earliest known unequivocal rhinocerotoid sheds new light on the origin of Giant Rhinos and phylogeny of early rhinocerotoids

**DOI:** 10.1038/srep39607

**Published:** 2016-12-21

**Authors:** Haibing Wang, Bin Bai, Jin Meng, Yuanqing Wang

**Affiliations:** 1 Key Laboratory of Vertebrate Evolution and Human Origins of Chinese Academy of Sciences, Institute of Vertebrate Paleontology and Paleoanthropology, Chinese Academy of Sciences, Beijing, 100044, China; 2 University of Chinese Academy of Sciences, Beijing, 100049, China; 3 Division of Paleontology, American Museum of Natural History, Central Park West at 79th Street, New York, NY 10024, USA

## Abstract

Forstercooperiines are a group of primitive rhinocerotoids with a relatively large body size in the Eocene, and normally considered to be closely related to Giant Rhinos. Here we report a new forstercooperiine, *Pappaceras meiomenus* sp. nov., from the late Early Eocene Arshanto Formation, Erlian Basin, Nei Mongol, China. *Pappaceras* is the earliest known unequivocal rhinocerotoid, and the holotype of the new species, represented by the most complete cranium of forstercooperiines known to date, shows the earliest evidence of reduction of the first upper premolar in rhinocerotoids, and resembles paraceratheriine *Juxia* in basicranial features, supporting the interpretation that the forstercooperiine clade is ancestral to paraceratheriines. The new species also displays some similarities with amynodontids in craniodental structures. Phylogenetic analysis identifies *P. meiomenus* as a basal taxon of the monophyletic forstercooperiines. It also reveals novel phylogenetic relationships of early rhinocerotoids that indicates *Uintaceras* is the sister group of paraceratheriids, to which amynodontids are more closely related than to any other group of rhinocerotoids. Furthermore, the eggysodontid clade is excluded from hyracodontids and placed as the sister group of rhinocerotids. Hyracodontidae, excluding paraceratheriids and eggysodontids, is placed as the most basal group of the rhinocerotoids.

The Eocene Epoch was the dawn of the age of modern mammals^1^, recording the first appearances of placental clades at the ordinal level, such as perissodactyls, artiodactyls, and primates. After its appearance during the early Eocene, perissodactyls became a dominant group by taxonomic richness with abundant fossils in the mammalian fauna in the middle Eocene^2^,^3^,^4^, then declined in the Oligocene during a period of climate change from the warm, humid and forestry Eocene to the dry, cool and open Oligocene. Thus, the Eocene is a crucial period for perissodactyl evolution, in which all main groups of perissodactyls have begun to be diversified since the beginning of the period. Among perissodactyls, Rhinocerotoidea, historically composed of Hyracodontidae (usually including paraceratheres), Amynodontidae and Rhinocerotidae, appeared in the later middle Eocene and was usually considered to be derived from the tapirmorph *Hyrachyus*^5^,^6^. The genus *Hyrachyus* is close to rhinocerotoids, but maintains a number of features primitively similar to basal tapirmorphs^7^. Although *Hyrachyus* was regarded as a rhinocerotoid genus in several recent studies^7^,^8^,^9^, this affinity can’t be warranted from inconsistent phylogeny^9^. Its taxonomy is strongly in need of revision, and whether it is a true rhinocerotoid still remains to be further explored in broader phylogenetic analyses. The early Eocene *Pataecops* and *Rhodopagus* were previously proposed to be closely related to hyracodontids, but their phylogenetic relationships remain controversial^10^,^11^. The earliest unequivocal rhinocerotoids from North America were those from the middle Eocene early Uintan deposits (Washakie B)^11^,^12^, where the earliest members of three families of Rhinocerotoidea, represented by *Triplopus, Amynodon*, and *Uintaceras*, appeared simultaneously. Similarly, the earliest known unequivocal rhinocerotoids from Asia, represented by *Tripolopus, Rostriamynodon*, and *Fostercooperia totadentata*, occurred nearly simultaneously in the early Middle Eocene Irdinmanhan, which is correlative to the North American Uintan^13^. However, one exception in the Asian record of rhinocerotoids is *Pappaceras*, which comes from the late Arshantan, normally correlative to the North American Bridgerian^13^. This genus represents the earliest known unequivocal rhinocerotoid at present.

One of the most noticeable issues in rhinocerotoid evolution has, since 1910 s, been the origination of Asian paraceratheres (Giant Rhinos), the largest terrestrial mammals ever to live on earth^11^,^14^. The finding of Eocene paraceratheriine *Juxia* made a remarkable breakthrough for this issue in 1964, suggesting that *Juxia* was ancestral to later giant rhinos^15^. At the same time, *Juxia* narrows the morphological gap between forstercooperiines and paraceratheriines, favoring the hypothesis of a paracerathere affinity of forstercooperiines, which was originally proposed by Wood^16^,^17^, and subsequently summarized by Radinsky^11^. Since then, forstercooperiines have commonly been regarded as related to paraceratheres, because of their palaeogeographical distribution and relatively large body size; this interpretation is generally accepted by most of authors. However, forstercooperiines remain one of the most poorly-known rhinocerotoids and consequently, comparisons reliant on incomplete craniodental specimens cloud its systematic position, for instance the eggysodontid affinity of *Pappaceras* recently proposed by Bai and Wang^18^. In addition, there seems some morphological gap, such as the absence of enlarged and spatulate I1/i1 in forstercooperiines, between the taxa in question based on the current collection, which is somewhat tricky to explain in comparative study.

The interrelationships of early rhinocerotoids still remain unclear with regard to the wastebasket-like hyracodontids and enigmatic amynodontids. Previous phylogenetic works on early rhinocerotoids were usually not extensive, of which only a few phylogenetic studies involved forstercooperiines, and this group was commonly regarded as either a primitive member or the sister group of paraceratheriids^7^,^19^,^20^, or nested within a polyphyletic assemblage of early non-hyracodontid rhinocerotoids^8^. The unstable phylogenetic placement of forstercooperiines may be attributed not only to incomplete morphological information of fragmentary fossils but also to the uncertainty of relationships among main groups of rhinocerotoids. Particularly, the hyracodontids is generally regarded as a wastebasket since Radinsky’s work^8^,^11^,^21^, which encompasses all non-amynodontid and non-rhinocerotid taxa from the Eocene to Oligocene of North America, Asia and Europe. Another important unsolved issue in the evolution of early rhinocerotoids is the relationship between amynodontids and other rhinocerotoids^22^. As such, the phylogenetic relationships of early rhinocerotoids remains to be resolved.

Although *Fostercooperia* and *Pappaceras* were described previously^16^,^17^,^19^,^23^, here we provide significant new cranial morphology of *Pappaceras* and demonstrate its appearance actually in the Arshanto Formation rather than the Irdinmanha Formation as previously suggested. Based on the new data, we support the validity of the genus *Pappaceras* Wood, 1963, which was widely considered as the synonym of *Forstercooperia*^11^,^19^, and as a result, the name of the type species *Pappaceras confluens* (=*Forstercooperia confluens* Lucas *et al*.^19^) is considered to be valid. In addition, a new combination *Pappaceras minuta* (=*Forstercooperia minuta* Lucas *et al*.^19^) becomes necessary. Morphologically, *Forstercooperia*, especially the type of species *F. totadentata*, is too poorly-known to encompass all specimens formerly assigned to *P. confluens* and *P. minuta*, which share more characters with each other than to *F. totadentata*. Moreover, our recent fieldwork clarifies the lithological units in the Huheboerhe area, Erlian Basin, which clearly shows that *Pappaceras (P. minuta* and *P. confluens*) is from the Arshanto Formation, which is stratigraphically below *F. totadentata* from the overlying Irdinmanha Formation^24^,^25^.

## Results

### Geological setting

**Figure 1:**
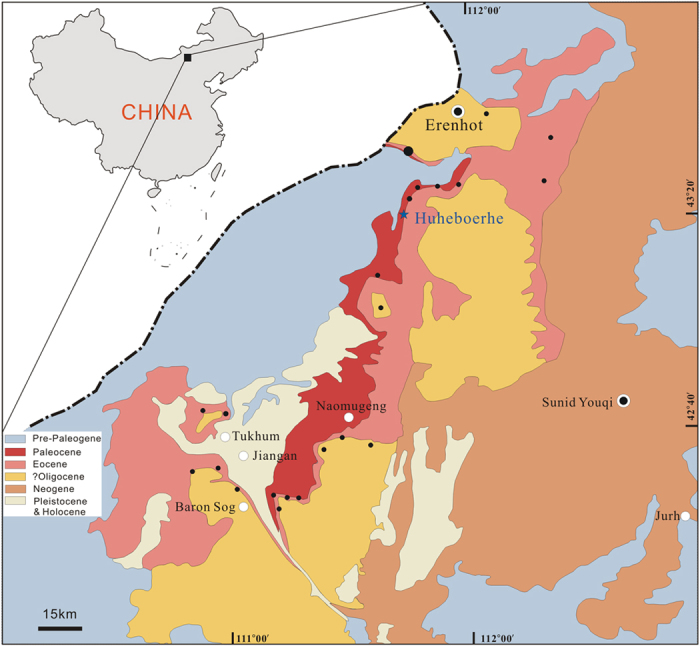
Location of the main fossil localities in the Erlian Basin. Blue solid star denotes the locality where IVPP V20254 was unearthed. Black solid circles mean other fossil localities of Erlian Basin, and no-solid circles denote towns and villages. *Scientific Reports* remains neutral with regard to jurisdictional claims in published maps. (The maps were created in Corel DRAW X3 (v. 13.0) by Haibing Wang).

**Figure 2:**
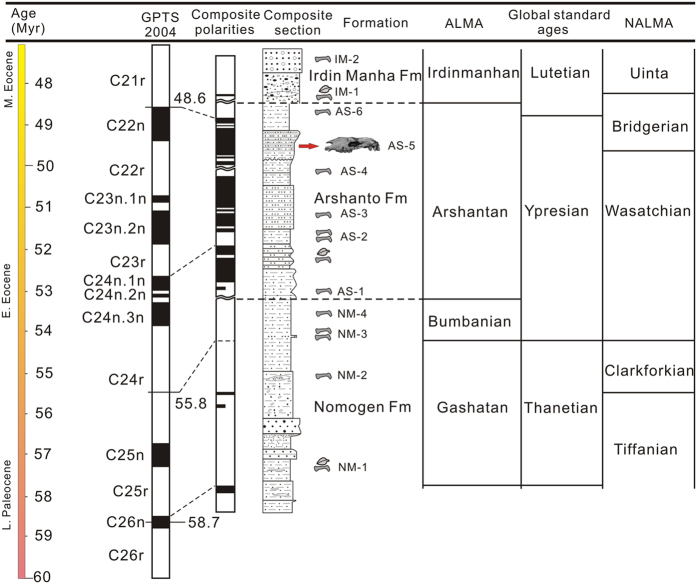
Palaeogene stratigraphy, palaeomagnetic polarities, mammalian horizons and their correlation in the eastern Erlian Basin (modified from previous ref. 25). The red arrow marks the bed (AS-5) in which the holotype specimen of *P. meiomenus* was discovered. (The figure was generated in Corel DRAW X3 (v. 13.0) by Haibing Wang).

The holotype specimen of the new species was collected from the upper part of the Arshanto Formation at Huheboerhe Area, Erlian Basin of Nei Mongol in north China (Fig. 1). The sediments, bearing the new specimen, are mainly grayish green, variegated sandy siltstone and fine sandstone^24^,^25^. Recent stratigraphic and palaeomagnetic studies demonstrate that Arshanto Formation is mainly Early Eocene in age rather than Middle Eocene, in contrast to previous convention (Fig. 2)^24^,^25^,^26^. The Arshantan Land Mammal Age is correlated to the middle Ypresian through the earliest Lutetian of the Geological Time Scale, and is also correlated with most of the Bridgerian of the North American Land Mammal Ages^25^ (Fig. 2). Accordingly, the age of the new specimen is estimated as late Early Eocene so that *Pappaceras* represents the earliest record of known unequivocal rhinocerotoid genus.

**Systematic palaeontology**

Class Mammalia Linnaeus, 1758

Order Perissodactyla Owen, 1848

Superfamily Rhinocerotoidea Gray, 1825

Family Paraceratheriidae Osborn, 1923

Subfamily Forstercooperiinae Kretzoi, 1940

*Pappaceras* Wood, 1963

Type species: *Pappaceras confluens* Wood, 1963

Include species: the type species, *Pappaceras minuta (Forstercooperia minuta*, Lucas *et al*.^19^) and *Pappaceras meiomenus* sp. nov.

Revised diagnosis: small forstercooperiine with medium body size. Skull length about 390 mm and length of M1-3/m1-3 about 70–90 mm. Skull dolichocephalic; premaxilla in contact with nasal and narial notch anteriorly ending above upper canine; preorbital fossa present, but relatively shallow. Large maxilla with a weak facial crest; infraorbital foreman situated above P4; zygomatic arch slender; frontal wide between orbital and followed by a distinct flange overhanging postorbital cavity. Postglenoid process massive, transversely broad, separated from posttympanic process to make external acoustic meatus open. Posttympanic process ventrally enlarged, partially coaslesced with paroccipital process; paroccipital process relatively slender and slightly longer than posttympanic process. Posttympanic-paroccipital foramen present. Sagittal crest distinct. Three upper/lower incisors conical and sub-equal in size with relatively tight arrangement; canines relatively stubby, larger than incisors. Diastema short. Upper premolars non-molarized and transversely wide with semi-circle lingual base; ribs of upper premolars distinct; reduction of P1 present in some species. metastyle of M2 elongated, metacone of M3 present and lingually oriented with a short metastyle. Lower premolars non-molarized; protolophid of lower molars relatively anterolingually extended. Differs from *Forstercooperia* in having sub-equal upper incisors, longitudinally relatively elongated upper canines, non-molarized upper premolars and a smaller size.

*Pappaceras meiomenus* sp. nov.

Holotype: a complete cranium (IVPP V20254). This specimen is now housed in the collections of Institute of Vertebrate Paleontology and Paleoanthropology (IVPP) at Chinese Academy of Sciences, Beijing, China.

Etymology: the specific name meiomenus after the Greek word μειωμένος, reduced, referring to reduction of upper premolars.

Diagnosis: medium-size species of *Pappaceras*. Length of upper molars series about 80 mm, skull length about 390 mm. P1 reduced; transverse lophs of P2-3 V-shaped; metaloph of P4 short and separated from protoloph by a distinct notch; protolophs of upper premolars (especially in P4) straight and not posteriorly extended at lingual side; hypocone of upper premolars not separated from protoloph. Antecrochet of M1 present; metacone of M3 distinct; metastyle of M3 relatively long.

Type locality and age: upper part of Arshanto Formation in Huheboerhe area, Erlian Basin (known as “Upper gray clays, ‘Irdinmanha Formation’, 11 km or 7 miles southwest from Camp Margetts” in previous references^19^,^24^,^25^), Nei Mongol, China; late Early Eocene.

## Description

### Cranium

**Figure 3:**
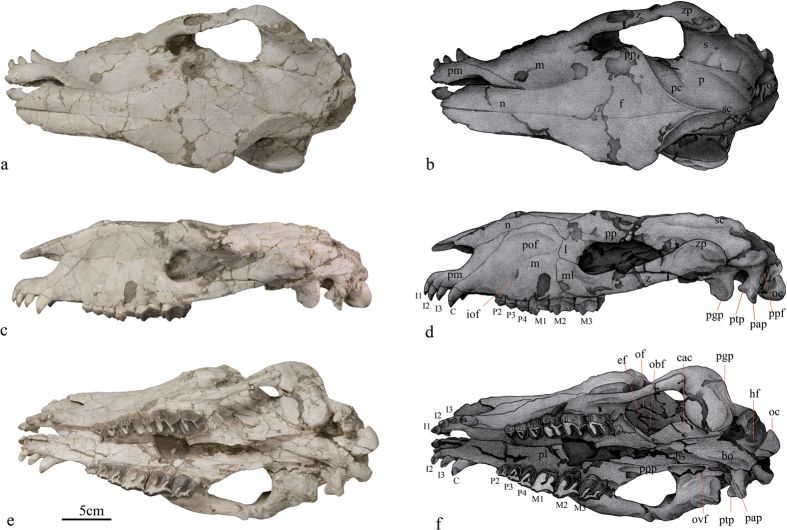
Cranium of *Pappaceras meiomenus*. Photographs in dorsal (**a**) lateral (**c**) and ventral (**e**) views, and corresponding interpretive drawings (**b**), (**d**,**e**) respectively. Abbreviation: bo, basioccipital; bs, basisphenoid; cac, caudal opening of alar canal; ef, ethmoid foramina; (**f**) frontal; hf, hypoglossal foramen; if, incisive foramen;; i of, infraorbital foramen; l, lacrimal; m, maxilla; ml, malar; n, nasal; o, occipital; oc, occipital condyle; of, optic foramen; obf, orbital foramen; ovf, oval foramen; p, parietal; pap, paroccipital process; pl, palatine; pc, postorbital crest; pgp, postglenoid process; pm, premaxilla; pof, preorbital foramen; ppf, posttympanic-paroccipital foramen; pp, postorbital process; ppp, pterygoid process of palatine; ptp, posttympanic process; s, squamosal; sc, sagittal crest; z, zygomatic; zp, zygomatic process of squamosal; C, upper canine; I1-3, the first to third upper incisor; P2-4, the second to fourth upper premolar; M1-3, the first to third upper molar.

The skull is typically dolichocephalic without horns. Its length is about 390 mm (Table 2 in Supplementary Information). The rostrum is unique, and the ascending part of the premaxilla contacts the nasal and extends posteriorly, forming a shallow narial notch at the level of upper canine. The horizontal part of the premaxilla represents an arc outline on ventral surface, and bears I1-3 relatively tightly arranged. The nasal, occupying the entire roof of the preorbital region, is thin and long with its cross section being plate-like. Anteriorly, the nasal is triangular with the apex extending above I2. Posteriorly, the nasal contacts frontal with a posteriorly concave suture. In ventral view, the palatine process of premaxilla is partially damaged and slightly compressed. The incisive foramen is probably paired on the account of the relatively curved medial margin of the horizontal part of premaxilla. The maxilla is large and nearly trapezoidal in lateral view, in which a shallow preorbital fossa is present. A sizeable infraorbital foreman is situated above the P4. The zygomatic is relatively short, and posteriorly, it contacts the zygomatic process of squamosal nearly dorsoventrally. The lacrimal has a large facial exposure that seems roughly quadrate. The fossa for the lacrimal sac is distinct and relatively laterally situated in the anterior part of orbital region. On the lateral wall of the palatine, the maxillary foramen is badly compressed and lateroventrally situated below the fossa for lacrimal sac, while the compressed sphenopalatine foramen and posterior palatine foramen are probably present, but are hardly visible. The zygomatic arch is distinctly slender and extends slightly laterally. The postorbital process of the zygomatic is weak and situated 40 mm behind the anterior border of the orbital on the right. The frontal forms a large part of the interorbital region, in which the dorsal surface is flat and roughly diamond-shaped in outline, and orbital part has a distinctly concave exposure. The supraorbital process of frontal is stout and rough on the surface. The frontal crest arises from about the postorbital process. These two crests are weak anteriorly, and gradually curve and thicken posteriorly, and at the level of glenoid cavity fuse into the sagittal crest. The lateral wall of the alisphenoid bears three foramina from above, ethmoid foramen, optic foramen, orbital foramen respectively. The opening of the foramen rotundum is probably separated by a thin plate from that of the foramen orbitale, but it is not clearly identified due to compression in this specimen. Medial to the postglenoid process is the oval foramen that is separated from the middle lacerate foramen. Lateral to the pterygoid, the posterior opening of the alar canal is situated about 40 mm anterior to the foramen oval. The external surface of parietal is narrow anteriorly and relatively wide posteriorly. The posterior part of parietal bears several nutrient foramens. The squamosal ventrally bears the glenoid cavity, consisting of anterior condyle and posterior fossa. The long axis of the condyle is slightly anterolaterally extended. The postglenoid process is rather massive, transversely broad and posterolaterally extends. It has two articular facets, facing anteriorly and laterally. Posterior to the postglenoid process, the postglenoid foramen is absent. The postglenoid process is posteriorly separated from the posttympanic process, making the external acoustic meatus widely open. The left petrosal is partially damaged and not distinctly exposed to identify specific characters, whereas the right is not preserved. Ventrally, the posttympanic process is enlarged in the ventral part with a globular outline. Laterally, the squamosal-temporal crest is sharp in the anterior portion and blunt posteriorly. The occipital is transversely relatively broad, and the occipital surface is nearly vertical, on which the protuberance is weak. The occipital lateroventrally bears the paroccipital process that is relatively slender and slightly longer than the posttympanic process. The paroccipital process is tightly attached to the enlarged posttympanic process, but does not completely coalesce with the latter. Lateral to the two processes, a foramen is at the furrow’s upper end (temporarily defined as posstympanic-paroccipital foramen) (Fig. 3). The occipital condyles are situated much more vertically rather than horizontally to confine an oval foramen magnum. Ventrally, the basilar tubercles are relatively well-developed and there is no distinct ridge on the ventral of basioccipital. The hypoglossal foramen is distinct and relatively far from the occipital condyle.

### Dentition

Upper incisors are well preserved and moderately worn, except for the right I1, which was lost but its alveolus is present. All the five preserved incisors are single-rooted, conical and sub-equal in size, extended anteriorly downward. Upper incisors are relatively curvedly and tightly arranged with 5mm diastema between I1 and I2, and I2 and I3. The I1 is more mesial than the others. The I2 and I3 have almost same size and both are slightly larger than I1. Each incisor has a rounded cross section, and its lingual crown facets are divided by a mesial ridge into an anterior and a posterior worn facet. The anterior one becomes progressively smaller from I1 to I3, while the posterior one is gradually larger. The upper incisors lack cingula. The canine is closely located to I3. It is stubby and larger than upper incisors. Only an oval wearing facet presents on its anterior crown. Posterolaterally, the tooth crown bears a weak ridge. Its root is more robust than those of upper incisors. The cingulum is absent. The left P1 is absent and there is no alveolus left, while the right one only preserves a single small root. The P2 is triangular in occlusal outline, with two transverse lophs forming a V-shaped outline. The protoloph is anterolabially extended from the protocone, contacting the ectoloph with a notch at the base of paracone. The metaloph is weaker than the protoloph and relatively transversely oriented. The parastyle of P2 is weak, and the rib is distinct on the labial wall of the paracone whereas less-developed of metacone. The cingulum is distinct at anterior, lingual and posterior base, but weak at the base of the protocone. The labial cingulum is less-developed and interrupted at the base of paracone. The posterior part of P2, especially the metacone, is more worn. The P3 is similar to P2 in basic structure, but has a larger size and a round rectangular outline in occlusal view. Its width is greater than the length. The parastyle is distinctly more developed than in P2, is somewhat pinched, anterolabially oriented, and separated from the paracone by a relatively deep parastyle fold. In the lingual side of the protocone there is a less-developed crista. The protoloph is less oblique and stronger than that of P2. The metaloph, slightly weaker than the protoloph, is convex posteriorly and joins the ectoloph between paracone and metacone in a high position. On the lingual side, the metaloph extends anterolingually and joins protocone. The lingual base of the crown is smoothly more semi-circular than that of P2. Compared with preceding premolars, The P4 has a more distinct parastyle, and deeper parastyle fold, a more distinct crista, and is relatively wider and narrower. The protoloph of P4 is much stronger than that of P2-3, while the metaloph is rather weak, short, transversely extended, and separated from the protoloph by a notch. The cingulum is similar to that of P2-3. The M1 is quadrate with distinct parastyle and parastyle fold on the buccal wall. The paracone rib is prominent, while the metacone rib is faint. A distinct antecrochet is present on the protoloph. The M2 is basically similar to M1, but differs from the latter in having a larger size, a more distinct parastyle, a deeper parastyle fold and a flatter labial surface of the metacone. Its ectoloph extends more lingually, and the metacone is much longer than those in M1 and M3. The M3 is relatively trapezoidal in outline with a distinct and lingually located metacone. The ectoloph does not completely align with the short metaloph, so that the metacone is very short. The cingulum of molars is distinct and continuous at bases of lingual, anterior and posterior side, while the buccal cingulum is rather weak. On the wear surface, the Hunter-Schreger Bands (HSB) are oriented transversely in the anterior dentition (upper incisors and canines) and vertically in the upper molars, similar to those of most rhinocerotoids^27^.

## Discussion

The well-preserved skull is unquestionably referable to Forstercooperiinae mainly for its cranial morphology (e.g. shallow nasal incision and distinct preorbital fossa) and unique anterior dentition (e.g. conical, sub-equal upper incisors and relatively stubby canines). We assign the new specimen to a new species of *Pappaceras* based on the stratigraphical evidence (same with *Pappaceras* rather than *Forstercooperia* in Arshantan age) and its characteristic dental morphology (see diagnosis). The comparisons of craniodental features between *Pappaceras meiomenus* and the earliest representatives of main groups of early rhinocerotoids, which were considered to be highly comparable as proposed by various authors^7^,^8^,^11^,^18^,^20^, are essential for the study of rhinocerotoid evolution.

*P. meiomenus* is evidently distinguishable from rhinocerotids by lacking characteristics of rhinocerotids, such as the tusk-like I1/i2, which occurs as an incipient condition later in the earliest unquestionable rhinocerotid *Teletaceras*^28^, Compared with *Juxia*, an ancestor to the later giant paraceratheriids, *P. meiomenus* differs by its smaller body size, shallow nasoincisor notch terminated above upper canines, nasal-premaxilla contact, relatively stubby upper canines, reduced upper premolars and distinct metacone of M3. Moreover, *P. meiomenus* is similar to *Juxia*, but distinguishable from other early rhinocerotoids (except for *Rostriamynodon* as discussed below) in following features: (1) relatively long basicranium^29^; (2) the posterior end of zygomatic plate-like and dorsoventrally compressed; (3) a posterior end of zygomatic arch forming lobe-shaped blade; (4) a shallow preorbital fossa; (5) a wide frontal between the orbital and followed by a flange overhanging the postorbital cavity; (6) incisors arranged in straight lines and weakly converging anteriorly; (7) ridge-like nuchal crest. The posttympanic process and paroccipital process of *Juxia* are more developed, wider, and thicker than those of *P. meiomenus*, however, and the latter began to show the rudimentary coalescence and enlargement of the former. Similarly, despite lacking the characteristics of amynodontid cheek teeth that possess straight ectolophs on upper molars and short premolar series, *P. meiomenus* shares the character (5), (6), (7) as listed above with the most primitive amynodontid *Rostriamynodon*^30^. Furthermore, it is interesting to note that the some features of *P. meiomenus* are reminiscent of those of amynodontids: a relatively large canine, distinctly reduced P1, a short postcanine diastema, P4 transversely wide with high and strong protoloph, weak and short metaloph (metaconules), and M3 quadrate in outline. The derived amynodontid *Sharamynodon* shows a similar condition to *P. meiomenus* and *Juxia* with coalesced and anteroposteriorly thick paroccipital and posttympanic processes, although the paroccipital process is more posteriorly extended. Generally, the younger group of eggysodontids are similar to forstercooperiines with short diastema and lingually extended protolophids of lower cheek teeth^18^,^31^. In spite that *P. meiomenus* is close to the Late Eocene eggysodontids *Proeggysodon* and *Guangnanodon* in size (based on length of lower molar series)^18^,^31^, eggysodontids differ from the latter by having complete upper premolars, large and nearly vertically oriented canines, primitively speculate incisors that vary in number (2 or 3) across different genera, and more molarized premolars^32^,^33^, and the known craniodental features of eggysodontids^34^ are more comparable to those of rhinocerotids rather than forstercooperiines. The enigmatic genus *Uintaceras* was considered as the sister group of rhinocerotids for its distinctive features of anterior dentition (buccolingually compressed upper incisors with a triangular profile) and characteristics of cheek teeth and postcranial elements^8^,^35^. Although Holbrook and Lucas^35^ pointed out some cranial differences between forstercooperiines and *Uintaceras*, it should be noted that the reconstruction of skull of *Uintaceras* was mainly based on laterally compressed and distorted materials of UCMP 69722 and UW 2410. Some characters used for distinguishing *Uintaceras* from fostercooperines show similarities in mentioned taxa, when compared to those of other early rhinocerotoids. For instance, both of them possess shallow nasal notches, high maxillae, prominent sagittal crests, the occipitals not strongly posteriorly inclined, and probably shallow maxilla fossae. In terms of dentitions, they are similar in having non-molarized premolars, presence of M3 metastyle and short diastema. It is interesting to mention that some similarities between *Uintaceras* and amynodontids in both dental and postcranial characters were mentioned, but considered as a result of scaling^35^. The most conspicuous feature that distinguishes *Uintaceras* from fostercooperines lays in its buccolingually compressed upper incisors with triangular profile. This specular feature, however, is even different from anterior teeth of all rhinocerotoids. It seems that *Uintaceras* is still more closely related to forstercooperiines and amynodontids than to rhinocerotids. In addition, the late Bridgerian *Hyrachyus princepts* (AMNH 12364)^36^ shows some similarities with either fostercooperines or *Uintaceras* in craniodental characters, but different from other species of *Hyrachyus* in having a flange posterior to the orbital, somewhat coalesced and enlarged posttympanic and paroccipital processes (with forstercooperiines), and especially relatively chisel-blade I1-2 (with *Uintaceras*). These similarities seem not unexpectable, since *Hyrachyus princepts*, the second largest species of *Hyrachyus* beside *H. grandis*, which was included in *Uintaceras*, was considered to give rise to the Uintan *H. grandis* by Wood^36^. However, the large parastyles of upper molars and relatively reduced cristid obliqua of lower molars displaced the species in the side of tapiroids rather than rhinocerotoids.

**Figure 4:**
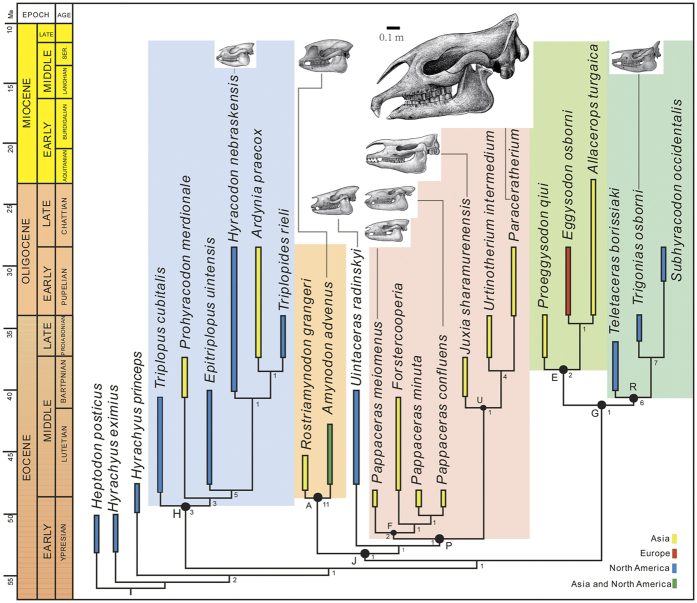
Correlation of geographical distributions and phylogenetic relationship of early rhinocerotoids based on the single most-parsimonious tree (tree length = 430, consistency index = 0.540, retention index = 0.704). For convenience, node A for Amynodontidae, E for Eggysodontidae, F for Forstercooperiinae, H for Hyracodontidae, P for Paraceratheriidae, R for Rhinocerotidae, U for Paraceratheriinae. Numbers by the nodes denote the Bremer values.

Phylogenetic analysis yielded one most-parsimonious tree (MPT) that is displayed in Fig. 4. Our phylogenetic analyses placed *Pappaceras meiomenus* as the most basal taxon of the monophyletic forstercooperiine clade (node F). The separate placement of *P. meiomenus* from the group of *P. confluens* and *P. minuta* implies that *P. meiomenus* represents an alternative evolutionary direction by the reduction of upper premolars in this genus. The monophyletic hyracodontids (node H), amynodontids (node A), paraceratheriids (node P), and rhinocerotids (node R) are also supported in the result of phylogenetic analyses. Despite the fact that only craniodental features are known for unequivocal forstercooperiines, the close affinity between forstercooperiines (node F) and paraceratheriines (node U) was supported in our results with solid evidence that consists of several synapomorphies (Supplementary Information, Table 1).

Our phylogenetic analysis reveals novel interrelationships of early rhinocerotoids (Fig. 4). Based on the node J, the clade comprising amynodontids, paraceratheriids and *Uintaceras* is monophyletic. The genus *Uintaceras*, which was considered as “North American forstercooperiines” and subsequently regarded as the sister group of rhinocerotids^35^, is closely related to the Asian paraceratheriid clade, suggesting that *Uintaceras* should be North American relatives of paraceratheriids. The similarities of incisors and postcranial elements between *Uintaceras* and rhinocerotids as previous proposed to favor the rhinocerotid affinity of *Uintaceras*, shows some parallelism in the evolution of early rhinocerotoids according to the result. Meanwhile, *Hyrachyus princeps*, possibly ancestral to *Uintaceras*, is positioned at the basal part of the present parsimonious tree, despite resembling *Uintaceras* in having uniquely buccolingually compressed I1 and distinctive flanges overhanging postorbital cavity. Interestingly, it is the amynodontid clade that is closely placed to paraceratheriids and North American *Uintaceras*, contrasting with the previous view that regarded amynodontids as a unique family divergent from other rhinocerotoids at early stage of rhinocerotoid evolution^7^,^10^,^37^. Particularly, the reduction of P1 makes *P. meiomenus* linked with amynodontids, for this feature is present in *P. meiomenus* as the earliest occurrence across rhinocerotoids, and characteristic of all amynodontids as well. The eggysodontid clade (node E) is revealed as the sister group of the rhinocerotid clade (node R), in contrast to the previous views of either paraceratheriines or forstercooperiines affinity^11^,^18^. The hyracodontid clade, is redefined as the most basal group of rhinocerotoids in our results, and comprises the primitive *Triplopus, Prohyracodon*, several derived types from North America (e.g. *Hyracodon* and *Triplopides*) and Asia (e.g. *Ardynia*) with small to medium body size and distinctively fast-running adaption of the skeletons^21^,^29^.

A parallel evolutionary trend is evident in the evolution of dental characters of early rhinocerotoids, particularly the anterior dentition and M3, which were generally regarded as the diagnostic features of the four main families of rhinocerotoids^5^,^6^,^21^. Large lower canines are present in forstercooperiines, amynodontids, and eggysodontids, of which the incisors are uniquely conical for the former and primitively spatulate for the latter two groups. On the other hand, the specialization of incisors is represented by tusk-like form for paraceratheriines and rhinocerotids with enlargement of I1/i1 and I1/i2 respectively, whereas it turns to be conical for forstercooperiines and North American hyracodontids. The lingually deflected metacone of M3, that was regarded as characteristic of hyracodontids^7^,^21^, is plesiomorphic for all clades except for eggysodontids, of which M3 specimen from Eocene is still unknown. Meanwhile, the homologies of cranial and postcranial characters, which were not extensively explored under the frame of phylogeny but equally significant as dental evolution, are revealed in the present results, for instance the hyracodontid clade is supported by 7 postcranial features, while 7 synapomorphies supporting the monophyly of amynodontid-paraceratheriid-*Uintaceras* clade are related to cranium.

Given the phylogenetic relationship of early rhinocerotoids, it reveals the primary trend of independent origination and evolution in different continents in relevant groups of paraceratheriids, amynodontids and hyracodontids. The origination and evolution of the paraceratheriid clade is definitely restricted into Asia, whereas its sister group *Uintaceras* appeared slightly later (Uintan) in North America than *Pappaceras* (Arshantan) in Asia, and like *Hyrachyus princeps*, evolved only in the North American continent. Likewise, the earliest records of amynodontids are represented by *Rostriamynodon* and its contemporary *Amynodon* occurring in Asia (Irdinmanhan) and North America (Uintan), respectively. *Triplopus*, the most primitive hyracodontid, first appeared in the Irdinmanhan and Uintan, and its descendants evolved independently to obtain the incisors with two different forms (spatulate and conical) in Asia and North America (the anterior dentition of *Prohyracodon* unknown). The distinctively cursorial adaptation of hyracodontids is relatively conservative from primitive Eocene *Triplopus* to derived Oligocene *Hyracodon*. By contrast, the eggysodontids and rhinocerotids seem to have arose independently in Asia and North America, respectively. The Asian origination of European eggysodontids is supported by the earliest occurrence in the Late Eocene of Asia, suggesting that this group dispersed into Europe after the “Grande Coupure”^18^. Given the presence of the earliest representative of rhinocerotids in North America^28^, as well as the poor record of Asian Eocene rhinocerotids and the systematically problematic status of those assigned specimens^29^,^38^,^39^, the North American continent should be central for rhinocerotids at early stage of the evolution. Another evident evolutionary trend is the maximum size of amynodontids and paraceratheriids, which is generally congruent with other Cenozoic large land mammals^40^. This pattern is greatly contributed to the niche expansion, in which *Juxia* evolved to the largest terrestrial land mammal *Paraceratherium* in paraceratheriids (Fig. 4), whereas *Rostriamynodon* turned to be the assumedly uniquely semi-aquatic *Metamynodon* in amynodontids^41^. On the other hand, the paraceratheriids, amynodontids and hyracodontids gradually faded away after the Oligocene epoch, but the rhinocerotid clade just began to diversify since the Late Eocene.

## Methods

We use capital I, C, P, M for upper incisors, canine, premolars, and molars respectively. For phylogenetic analyses, we created a new data matrix, which includes 25 taxa, and 203 characters. The phylogenetic analyses were performed using TNT 1.1 with a traditional research method^42^, 1000 replications and the trees-bisection-reconnection branch-swapping algorithm (TBR) applied in our analyses. All characters are equally weighted, and all characters except for character 29 and 30 are non-additive. Gaps are treated as “missing” and multistate taxa interpreted as polymorphism. The analyses yielded one parsimonious tree that is presented in Fig. 4. Other results (character list and data matrix) are presented in the Supplementary Information (SI). The institution abbreviations used in text: IVPP, Institute of Vertebrate Paleontology and Paleoanthropology, Beijing, China; AMNH, American Museum of Natural History, New York, USA; UCMP, University of California Museum of Paleontology, Berkeley, USA; UW, University of Wyoming, Laramie, USA.

## Additional Information

**How to cite this article**: Wang, H. *et al*. Earliest known unequivocal rhinocerotoid sheds new light on the origin of Giant Rhinos and phylogeny of early rhinocerotoids. *Sci. Rep.* **6**, 39607; doi: 10.1038/srep39607 (2016).

**Publisher's note:** Springer Nature remains neutral with regard to jurisdictional claims in published maps and institutional affiliations.

## Supplementary Material

Supplementary Information
