# Supplementary material for: Earliest known unequivocal rhinocerotoid sheds new light on the origin of Giant Rhinos and phylogeny of early rhinocerotoids
Source: Sci Rep. 2016 Dec 21;6:39607. doi: 10.1038/srep39607 (PMC5175171; doi:10.1038/srep39607)
Supplement: Supplementary Information [file srep39607-s1.pdf]

Supplementary Information

Earliest unequivocal rhinocerotoid sheds new light on the origin of Giant Rhinos and phylogeny of early rhinocerotoids

Author: Haibing Wang[1,2], Bin Bai[1], Jin Meng[1,3], Yuanqing Wang[1*]

[1] Key Laboratory of Vertebrate Evolution and Human Origins of Chinese Academy of Sciences, Institute of Vertebrate Paleontology and Paleoanthropology, Chinese Academy of Sciences, Beijing, 100044, China.
[2] University of Chinese Academy of Sciences, Beijing, 100049, China.
[3] Division of Paleontology, American Museum of Natural History, Central Park West at 79th Street, New York, NY 10024, USA.

E-mail: wangyuanqing@ivpp.ac.cn

1. Character List

The characters used for our phylogenetic analysis are 203, 49 for cranium, 16 for mandible, 73 for teeth, and 65 for postcranial skeleton. Among, 131 characters are new, and 72 characters are directly adopted or modified from previous phylogenetic analysis studies, of which 23 characters are used here with different character states from original versions. The modified characters are mostly from Antoine et al. (2010), which focused on the phylogenetic interrelationships of highly diversified and derived rhinocerotids. In most cases, the multiple states (four or more) of modified characters were cut or combined to be two or three states, so that the character matrix can be more compact and accurate.

Cranium

(1) Nasal notch =0, above C; 1, above diastema; 2, above premolars. Corresponds to character 3 of Antoine et al. (2010), with different states.
(2) Nasal/lacrymal: contact = 0, long; 1, reduced or absent. Corresponds to character 6 of Antoine et al. (2010), with different states.
(3) Nasal bones: rostral end = 0, narrow; 1, very narrow. Corresponds to character 24 of Antoine et al. (2010), with different states.
(4) Nasal bones, length before notch = 0, short; 1, long. Corresponds to character 26 of Antoine et al. (2010), with different states.
(5) Premaxilla/nasal, contact = 0, present; 1, absent. Corresponds to character C3 of Holbrook (1999), with different states.
(6) Horizontal branch of premaxilla, extending direction = 0 horizontal; 1 downward.
(7) Preorbital fossa = 0, absent; 1, shallow; 2, deep. Corresponds to character C6 of Holbrook (1999), with different states.

(8) Premaxilla, horizontal branch = 0, short; 1, long.
(9) Premaxilla, horizontal branch = 0, slender; 1, stout, laterally expanded; 2, stout, laterally compressed.
(10) Incisive foramen = 0, paired; 1, single. Corresponds to character C5 of Holbrook (1999)
(11) Orbit: anterior border = 0, above P4–M1; 1, behind M2. Corresponds to character 7 of Antoine et al. (2010), with different states.
(12) Maxillary: anterior base of the zygomatic process of maxilla = 0, low; 1, high. Corresponds to character 10 of Antoine et al. (2010), with different states.
(13) Zygomatic arch = 0, low; 1, high. Corresponds to character 11 of Antoine et al. (2010), with different states.
(14) Zygomatic arch: postorbital process = 0, on jugal; 1, on squamosal. Corresponds to character 13 of Antoine et al. (2010).
(15) Zygomatic arch, lobe-shaped blade = 0, absent; 1, present.
(16) Secondary palatine = 0, flat; 1, concave.
(17) Skull: dorsal profile = 0, flat; 1, posteriorly raised. Corresponds to character 15 of Antoine et al. (2010), with different states.
(18) Squamosal: area between temporal and nuchal crests = 0, flat; 1, depression. Corresponds to character 17 of Antoine et al. (2010).
(19) External auditory pseudomeatus = 0, open; 1, partially closed. Corresponds to character 18 of Antoine et al. (2010), with different states.
(20) Occipital side = 0, slightly inclined forward or nearly vertical; 1, inclined backward. Corresponds to character 19 of Antoine et al. (2010), with different states.
(21) Skull = 0, dolichocephalic; 1, brachycephalic. Corresponds to character 23 of Antoine et al. (2010).
(22) Frontal, transverse width = 0, relatively narrow; 1, expanded.
(23) Frontal-parietal = 0, sagittal crest; 1, close frontoparietal crests; 2, distant crests. Corresponds to character 35 of Antoine et al. (2010).
(24) Occipital crest = 0, concave; 1, straight. Corresponds to character 36 of Antoine et al. (2010), with different states.
(25) Squamosal: articular tubercle = 0, smooth; 1 high. Corresponds to character 39 of Antoine et al. (2010).
(26) Squamosal: postglenoid process = 0, transversely wide; 1, transversely narrow.
(27) Basioccipital: basilar process = 0, smooth; 1, with a blunt median ridge; 2, with a sharp median crest.
(28) Squamosal: posterior groove on the zygomatic process = 0, absent; 1, present. Corresponds to character 45 of Antoine et al. (2010).
(29) Squamosal-occipital: posttympanic process and paraoccipital process = 0, separated; 1, partially coalesced; 2, completely coalesced. (adopted from Qiu and Wang, 2007)
(30) Squamosal: posttympanic process = 0, slender or flat; 1, ventrally thickened; 2, entirely thickened. (adopted from Qiu and Wang, 2007)
(31) Occipital: paraoccipital process = 0, slender; 1, robust. Corresponds to character

48 of Antoine et al. (2010).
(32) Occipital: paraoccipital process = 0, posteroventrally extended; 1, ventrally extended.
(33) Occipital: foramen magnum = 0, circular; 1, subtriangular. Corresponds to character 49 of Antoine et al. (2010).
(34) Basioccipital: median ridge on the condyle = 0, absent; 1, present. Corresponds to character 50 of Antoine et al. (2010).
(35) Postglenoid foramen = 0, present; 1, absent. Corresponds to character C10 of Holbrook (1999).
(36) Anterior face of postglenoid process = 0, flat or concave and undivided; 1, convex with medial ridge. Corresponds to character C12 of Holbrook (1999).
(37) Posttympanic process, ventral extension relative to paroccipital process, = 0, short; 1, nearly equal.
(38) Postglenoid process to occipital condyle, length, relative to the length of post-P1 portion= 0, short (<1/5); 1 long (>1/5). (adopted from Qiu and Wang, 2007)
(39) Sagittal crest = 0, ventrodorsally low; 1 ventrodorsally high.
(40) Supraorbital foramen = 0, absent; 1, present.
(41) Foramen ovale and foramen lacerium medium = 0, separated; 1, fused.
(42) Postglenoid process, facing direction of articular facet = 0, anterior; 1, anterolateral; 2, nearly lateral.
(43) Posttympanic process and Paroccipital process = 0, relatively slender; 1, transversely wide; 2, anteroposteriorly elongated.
(44) Paraoccipital-posttympanic foramen = 0, absent; 1, present.
(45) Position of the paraoccipital process related to posttympanic process = 0, posteromedially; 1, posteriorly.
(46) Temporal condyle, orientation = 0, anterolaterally inclined; 1, transversely extended.
(47) Maxilla =0, low; 1, high.
(48) Palatine, mesial ridge on the ventral surface = 0, absent; 1, present.
(49) Pterygoid fossa = 0, absent; 1, present

Mandible

(50) Symphysis: transverse constriction = 0, absent; 1, distinct. New character.
(51) Symphysis = 0, anterodorsally upraised; 1, nearly vertically upraised; 2, nearly horizontally upraised.
(52) Symphysis = 0, spindly; 1, anteroposteriorly constricted. Corresponds to character 54 of Antoine et al. (2010), with different states.
(53) Symphysis: posterior margin = 0, in front of p2; 1, level of p2–4. Corresponds to character 55 of Antoine et al. (2010).
(54) Diastema between lower incisors and lower premolars, length relative to cheek teeth series = 0, long (>1/5); 1, short (< 1/5).
(55) Diastema: depression between lower incisors and lower premolars = 0, absent; 1, present.

(56) Mental foramen = 0, in front of p1; 1, level of p2–4. Corresponds to character 56 of Antoine et al. (2010).
(57) Mandible: lingual groove = 0, present; 1, absent. Corresponds to character 57 of Antoine et al. (2010).
(58) Mandible: base = 0, straight; 1, convex. Corresponds to character 59 of Antoine et al. (2010), with different states.
(59) Ramus = 0, vertical; 1, inclined forward. Corresponds to character 60 of Antoine et al. (2010), with different states.
(60) Ramus: coronoid process = 0, well developed; 1, little developed. Corresponds to character 61 of Antoine et al. (2010).
(61) Mandibular Foramen = 0, below the teeth neck; 1, above the teeth neck. Corresponds to character 62 of Antoine et al. (2010).
(62) Postcotyloid process of dentary = 0, absent; 1, present. Corresponds to character C14 of Holbrook (1999).
(63) Mandible angle, form = 0, posteriorly extended; 1, rounded.
(64) Coronoid process, height relative to the mandible length = 0, high (>1/2); 1, low (<1/2).
(65) Coronoid process, form = 0, posteriorly curved; 1, vertical.

Teeth

(66) Length of upper premolars, relative to length of upper molars, LUP/LUM = 0, more than 0.5; 1, less than 0.5.
(67) I1: shape of the crown = 0, speculate; 1, conical; 2, buccolingually compressed.
(68) I1= 0, incisor-like; 1, tusk-like.
(69) I2 = 0, present; 1, absent. Corresponds to character 73 of Antoine et al. (2010).
(70) I2, shape of crown = 0, speculate; 1, conical; 2, buccolingually compressed.
(71) I3 = 0, present; 1, absent. Corresponds to character 74 of Antoine et al. (2010).
(72) I3 = 0, spetulate; 1, conical; 2, buccolingually compressed.
(73) Upper incisors, arrangement, = 0, arch; 1, anteroposteriorly straight.
(74) Upper incisors, size = 0, equal or subequal; 1, distinctly different.
(75) C1 = 0, present; 1, absent. Corresponds to character 75 of Antoine et al. (2010).
(76) C1 = 0, slightly larger than incisors, no specialization; 1, incisor-like, reduced; 2, enlarged.
(77) i1: crown = 0, developed, with a pronounced neck; 1, reduced. Corresponds to character 77 of Antoine et al. (2010).
(78) i1: orientation = 0, anterodorsally upraised; 1, nearly vertically upraised; 2, anteriorly or procumbent extended.
(79) i1: = 0, incisor-like; 1, tusk-like.
(80) i1: shape of crown= 0, speculate; 1, conical; 2, buccolingually compressed.
(81) i2 = 0, present; 1, absent. Corresponds to character 78 of Antoine et al. (2010).
(82) i2: shape = 0, incisor-like; 1, tusk-like. Corresponds to character 79 of Antoine et al. (2010).
(83) i3 = 0, present; 1, absent. Corresponds to character 81 of Antoine et al. (2010).
(84) Lower incisors = 0, tightly spaced; 1, loosely spaced.

(85) Lower incisors, arrangement, = 0, arch; 1, nearly anteroposterior.
(86) Lower incisors, size = 0, equal or subequal; 1, distinctly different.
(87) c1 = 0, present; 1, absent. Corresponds to character 82 of Antoine et al. (2010).
(88) c1: = 0, slightly larger than incisors, no specialization; 1, incisor-like, reduced; 2, enlarged.
(89) c1: shear facet with upper canine = 0, posterior; 1, anterior.
(90) c1, diastema between lower canine and the last lower incisor = 0, very short or absent; 1, long.
(91) P2–4: crochet = 0, absent; 1, present. Corresponds to character 84 of Antoine et al. (2010), with different states.
(92) Upper premolar: ribs on buccal wall = 0, distinct; 1, weak.
(93) Upper premolars: parastyle fold = 0, distinct; 1, weak.
(94) Upper premolars: form from P2 to P4 = 0, more molarized posteriorly; 1, less molaried posteriorly. (adopted from Qiu and Wang, 2007)
(95) P2–4: postfossette = 0, narrow; 1, wide. Corresponds to character 89 of Antoine et al. (2010), with different states.
(96) P1 = 0, present; 1, reduced or occasionally present; 2, entirely absent.
(97) P1, root = 0, not single; 1, single.
(98) P2, long axis oriented = 0, nearly transversely; 1, posterolingually.
(99) P2, size relative to P3-4 = 0, slightly small; 1, distinctly small.
(100) P2: protocone and hypocone = 0, completely fused; 1, slightly separated; 2, almost separated with a lingual bridge; 3, separated.
(101) P2: transverse lophs = 0, not jointed, weak; 1, jointed, V-shaped; 2, separated, well-developed.
(102) P3: protocone and hypocone = 0, completely fused; 1, slightly separated; 2, almost separated with a lingual bridge; 3, separated.
(103) P3: hypocone and metacone = 0, separated; 1 jointed.
(104) P4: protocone and hypocone = 0, completely fused; 1, slightly separated; 2, almost separated with a lingual bridge; 3, separated.
(105) P4: hypocone and metacone = 0, separated; 1 joinetd.
(106) P4, extension of protoloph at lingual side = 0, not posterior; 1, distinct posterior.
(107) P4: width/length = 0, less than 1.5; 1, 1.5 or more.
(108) P4: lingual bases, shape = 0, nearly triangle; 1, semi-circle; 2, straight.
(109) P2–4: crista = 0, absent; 1, present.
(110) Upper molar: parastyle, size = 0, large; 1, small. Corresponds to character D8 of Holbrook (1999).
(111) Upper molar: parastyle fold = 0, strong; 1, weak.
(112) (120)M1–2: metastyle = 0, short; 1, long. Corresponds to character 120 of Antoine et al. (2010).
(113) M1–2: metaloph = 0, long; 1, short. Corresponds to character 121 of Antoine et al. (2010).
(114) M1–2: posterior part of the ectoloph = 0, concave; 1, straight. Corresponds to character 122 of Antoine et al. (2010).
(115) M1: antecrochet = 0, absent; 1, present.

(116) M1: crochet = 0, absent; 1, present.
(117) M1: crista = 0, absent; 1, present.
(118) M2: antecrochet = 0, absent; 1, present.
(119) M2: crochet = 0, absent; 1, present.
(120) M2: crista = 0, absent; 1, present.
(121) M2: postfossette = 0, narrow; 1, wide.
(122) M3: metacone = 0, present; 1, absent.
(123) M3: metacone, location =0, laterally located; 1, lingually deflected.
(124) M3: ectoloph and metaloph = 0, distinct; 1, not fused, but confluent; 2, completely fused (ectometaloph). Corresponds to character 133 of Antoine et al. (2010), with different states.
(125) Lower cheek teeth: crown outline = 0, dialoph-shaped; 1, U/L-shaped.
(126) Lower cheek teeth: crown elongation = 0, absent; 1, present.
(127) p1 = 0, present; 1, reduced or occasionally present; 2, entirely absent.
(128) p1, root = 0, double; 1, single.
(129) p2: metalophid, extended direction = 0, postero-ligual; 1, transverse.
(130) p3: trigonid, length of paralophid = 0, short; 1, long.
(131) p3: talonid, entoconid = 0, absent; 1, isolated; 2, connected with hypoconid.
(132) p3: talonid, entolophid = 0, weak; 1, well-developed.
(133) p4: trigonid, length of paralophid = 0, short; 1, long.
(134) p4: talonid, entoconid = 0, absent; 1, isolated; 2, connected with hypoconid.
(135) p4: talonid, entolophid = 0, weak; 1, well-developed.
(136) Lower molars: protolophid, extending direction = 0, slightly lingual; 1, anteroposterior; 2, distinctly lingual.
(137) Lower molars: metalophid, extended direction = 0, nearly transverse; 1, distinctly postero-lingual.
(138) m3: hypoconulid = 0, present; 1, absent. Corresponds to character D14 of Holbrook (1999), with different states.

Postcranial skeleton
(139) Atlas: outline of the rachidian canal = 0, bulb; 1, mushroom. Corresponds to character 183 of Antoine et al. (2010).
(140) Atlas: alar notch = 0, shallow; 1, deep (end of notch is internally over the lateral edge of atlas alar). (adopted from Qiu and Wang, 2007)
(141) Atlas: height relative to length = 0, larger; 1, smaller.
(142) Scapula = 0, scapula-shaped or slightly elongated (L/W less than 2.0); 1, very elongated (L/W more than 2.0). Corresponds to character 190 of Antoine et al. (2010), with different states.
(143) Scapula, spine at distal end = 0, high; 1, low. Corresponds to the first character of Holbrook and Lucas (1997).
(144) Scapula, proximal ridge = 0, straight; 1, arched.
(145) Scapula, spinal tuberosity =0, weak; 1, well-developed.
(146) Scapula, scapular tuber = 0, well-developed; 1, weak.
(147) Scapula, acromion = 0, well-developed; 1, weak. Corresponds to the second

character of Holbrook and Lucas (1997).

(148) Humerus: deltopectoral crest = 0, weak; 1, well-developed.

(149) Humerus: caput = 0, almost rounded; 1, anteroposteriorly compressed; 2, lateromesially compressed.

(150) Humerus: distal articulation = 0, egg cup (shallow median constriction); 1, diabolo (deep median constriction). Corresponds to character 194 of Antoine et al. (2010).

(151) Humerus: median and lateral condyles, crest = 0, absent; 1 present.

(152) Humerus: distal gutter on the epicondyle = 0, absent; 1, present. Corresponds to character 196 of Antoine et al. (2010).

(153) Humerus: supinator crest = 0, weak; 1, prominent, extending proximally. Corresponds to the fifth character of Holbrook and Lucas (1997).

(154) Radius: length relative to the humerus = 0, short; 1, long.

(155) Radius: proximal and distal, mesolaterally expansion: 0, absent; 1, present.

(156) Radius: distal, articular facet for cuneiform: 0, present; 1, absent.

(157) Radius: proximal, articular facet for humerus: 0, deep; 1, shallow.

(158) Radius: facet with scaphoid = 0, rounded; 1, rhomboidal. Corresponds to the sixth character of Holbrook and Lucas (1997).

(159) Ulna: length relative to the humerus: 0, short; 1, long.

(160) Ulna: notch, distal facet = 0, narrow; 1, wide.

(161) Scaphoid: width relative to height = 0, small; 1 large.

(162) Scaphoid: proximal facet, outline = 0, quadrangle; 1, triangle or pentagon.

(163) Scaphoid: posteroproximal facet with semilunate = 0, present; 1, absent or contact. Corresponds to character 207 of Antoine et al. (2010).

(164) Scaphoid: trapezium-facet = 0, large; 1, small. Corresponds to character 208 of Antoine et al. (2010).

(165) Scaphoid: trapezium-facet, location = 0, ventral; 1, posterior.

(166) Semilunate: width relative to height = 0, nearly equal; 1, distinctly small.

(167) Semilunate: ulna-facet = 0, absent; 1, present. Corresponds to character 211 of Antoine et al. (2010).

(168) Semilunate: distal border of anterior side = 0, acute; 1, rounded. Corresponds to character 212 of Antoine et al. (2010).

(169) Cuneiform: distal facet for semilunate = 0, symmetric; 1, asymmetric; 2, L-shaped. Corresponds to character 214 of Antoine et al. (2010).

(170) Cuneiform: facet with radius = 0, absent; 1, present.

(171) Cuneiform: outline = 0, trapezoidal; 1, triangle. Corresponds to the seventh character of Holbrook and Lucas (1997).

(172) Magnum: posterior tuberosity = 0, short; 1, long. Corresponds to character 220 of Antoine et al. (2010).

(173) Magnum: facet with McII = 0, present; 0, absent.

(174) Unciform: McV-facet = 0, large; 1, small.

(175) Unciform: magnum-facet = 0, small; 1, large.

(176) McIII: length relative to proximal width = 0, 4-5 times; 1, more than 5 times; 2, less than 4 times. (adopted from Qiu and Wang, 2007)

(177) McIII: proximal, widen laterally= 0, absent; 1, present. New character.
(178) McIV: length relative to proximal width = 0, robust (less than 5 times); 1, slender (more than 5 times).
(179) McIV: McV-facet =0, large; 1, small. New character.
(180) McV: 0, functional; 1, vestigial. Corresponds to character 231 of Antoine et al. (2010).
(181) Femur: distal trochlea = 0, deep; 1, shallow.
(182) Femur: height, head relative to the great trochanter =0, equal; 1, lower; 2, higher.
(183) Femur: intercondyloid fossa = 0, narrow; 1, wide.
(184) Femur: neck = 0, well-developed; 1, weak.
(185) Femur: lesser trochanter = 0, well-developed; 1, weak.
(186) Patella: = 0, relatively thin; 1, anteroposteriorly thickening.
(187) Patella: outline, height, relative to width = 0, larger; 1, nearly equal or less.
(188) Patella: outline = 0, unflattened; 1, flattened. Corresponds to the ninth character of Holbrook and Lucas (1997).
(189) Tibia: lateral intercondyloid eminence, relative to the medial intercondyloid eminence = 0, equal in height; 1, higher.
(190) Astragalus: (transverse diameter/height) ratio = 0, TD/H less than 1.0; 1, TD/H more than 1.0. Corresponds to character 252 of Antoine et al. (2010), with different states.
(191) Astragalus: trochlea, medial groove = 0, deep; 1, shallow.
(192) Astragalus: distal cuboid-facet in anterior view = 0, narrow; 1, wide.
(193) Astragalus: tuberosity of the distal end at the medial side = 0, distinct; 1, weak.
(194) Astragalus: neck = 0, long; 1, short.
(195) Calcaneum: calcaneus tuber = 0, massive; 1, slender. Corresponds to character 266 of Antoine et al. (2010).
(196) Calcaneus: position of sustentaculum process = 0, low; 1, high.
(197) Calcaneus: fibula-facet = 0, absent; 1, present.
(198) Entocuneiform: = 0, separated with MtI; 1, fused with MtI; 2, reduced.
(199) Ectocuneiform: MtIV-facet = 0, absent; 1, present.
(200) MtIII: cuboid-facet = 0, absent; 1, present. Corresponds to character 275 of Antoine et al. (2010).
(201) MtIV: ectocuneiform-facet = 0, present; 1, absent.
(202) Limbs = 0, slender; 1, rather slender; 2, robust. Corresponds to character 279 of Antoine et al. (2010), with different ststes.
(203) Manus: = 0, tetradactyl; 1, tridactyl. Corresponds to the eighth character of Holbrook and Lucas (1997).

2. Data Matrix

Taxa	0000000001<br>1234567890	11111 11112<br>1234567890	2222222223<br>1234567890	3333333334<br>1234567890	4444444445<br>1234567890
Heptodon posticus	**1000000000**	**0000000000**	**0000000000**	**0000000000**	**0000000000**
Hyrachyus eximius	**0000??0000**	**0110000010**	**0000000000**	**0000?00?00**	**0000000000**
Hyrachyus princepts	**1000000000**	**0000000000**	**0100001011**	**0000000000**	**010100100?**
Rostriamynodon grangeri	**1000001010**	**0000010000**	**0101001???**	**0??0?1?011**	**011??01010**
Amynodon_advenus	**1000002010**	**0000010000**	**0101001000**	**01?0111011**	**011110101?**
F. totadentata	**0000001010**	**?????1????**	**??????????**	**??????????**	**??????10?1**
Pappaceras confluens	**0000001010**	**00001?0?00**	**010???????**	**????10??10**	**??????10?1**
Pappaceras minuta	**0000001010**	**0000110000**	**010100?0??**	**??0?10?110**	**01???01001**
Pap. meiomenus	**0000001010**	**00001?0000**	**010?000011**	**0100100110**	**012110100?**
Triplopus cubitalis	**100000000?**	**00000?0000**	**0000?0??00**	**00000?0000**	**?000000000**
H. nebraskensis	**1011000000**	**001?001000**	**0000100?00**	**0000000000**	**?100000110**
Pro. meridionale	**??????????**	**??????????**	**??????????**	**??????????**	**??????????**
Epitriplopus uintensis	**??10???00?**	**????0?????**	**??????????**	**??????????**	**?????????0**
Triplopides rieli	**1????0?00?**	**0?110??00?**	**?????1??00**	**00??0000??**	**?10?0000??**
Ardynia praecox	**11???00000**	**1001001?0?**	**00??01????**	**0?0????0?0**	**?1??0?0?10**
Eggysodon osborni	**??????????**	**??????????**	**??????????**	**??????????**	**??????????**
Allacerops turgaica	**??????????**	**??????????**	**??????????**	**??????????**	**??????0???**
Proeggysodon qiui	**??????????**	**??????????**	**??????????**	**??????????**	**?????????0**
J. sharamurenensis	**2001101110**	**00011?0000**	**0101001022**	**1100101110**	**0121101000**
U. intermedium	**??????????**	**??????????**	**??????????**	**??????????**	**?????????0**
Par. grangeri	**2111111121**	**1111101100**	**1101101022**	**11?0111110**	**0121101000**
Uintaceras radinskyi	**20??001???**	**100???0000**	**010100??10**	**01?1110010**	**?101001?0?**
Teletaceras borissiaki	**11111?0???**	**0000001000**	**0010012000**	**0001110000**	**0200010000**
Trigonias osborni	**2111100111**	**0010001010**	**0010012000**	**0011110000**	**0200010000**
S. occidentalis	**2111100111**	**0010001010**	**0020012100**	**0001110000**	**0200010000**

**Taxa**	**5555555556<br>1234567890**	**6666666667<br>1234567890**	**7777777778<br>1234567890**	**8888888889<br>1234567890**	**0000000001<br>9999999990<br>1234567890**
Heptodon posticus	**000000?000**	**000000000?**	**0000000000**	**0000000000**	**0000000000**
Hyrachyus eximius	**000?000000**	**?00000000?**	**0000000000**	**0000000000**	**0000000000**
Hyrachyus princepts	**??0?110100**	**0000002000**	**000000????**	**??????????**	**0000000000**
Rostriamynodon grangeri	**001001?000**	**?00??1?00?**	**0100020000**	**0000000210**	**000012?110**
Amynodon_advenus	**?????1?00?**	**?0???11001**	**0100020000**	**0000000210**	**010012?110**
Forstercooperia totadentata	**0101100000**	**0011001001**	**0100020000**	**0000000200**	**0001100001**
Pappaceras confluens	**0101100000**	**0011001001**	**0100020000**	**0000000200**	**0001100000**
Pappaceras minuta	**0101100000**	**0011001001**	**0100020000**	**0000000200**	**0001100000**
Papaceras meiomenus	**??????????**	**????01001**	**010002????**	**???????2??**	**0001111100**
Triplopus cubitalis	**00000?0010**	**?00000???0**	**????0????0**	**??????0000**	**0000000000**
Hyracodon nebraskensis	**00000?0110**	**?000001001**	**0100010001**	**0001000100**	**011000?003**
Prohyracodon meridionale	**??????????**	**?????0????**	**??????????**	**??????????**	**010000??00**
Epitriplopus uintensis	**00000?????**	**?????0????**	**??????0000**	**0001000100**	**0110000003**
Triplopides rieli	**00000??110**	**?000000000**	**0000010000**	**0001000100**	**011002?003**
Ardynia praecox	**0000010110**	**?0???00000**	**0000010000**	**0001000100**	**011002?003**
Eggysodon osborni	**1101010110**	**?0000010?1**	**0??00?0100**	**0010000200**	**0111010002**
Allacerops turgaica	**110101?1??**	**?0???010??**	**???00?0100**	**0000000200**	**0?11?10002**
Proeggysodon qiui	**110101?1??**	**??????????**	**??????0100**	**0010000200**	**??????????**
Juxia sharamurenensis	**0001010000**	**0011001001**	**0111010001**	**0001110110**	**0011100002**
Urtinotherium intermedium	**201101?000**	**?0111011?1**	**??????0211**	**0001110111**	**011??0???2**
Paraceratherium grangeri	**201001?000**	**?01000111?**	**1?111?0211**	**1?11111???**	**0111100002**
Uintaceras radinskyi	**0?0000?01?**	**?110?02002**	**020?00???2**	**??????0???**	**0001100000**
Teletaceras borissiaki	**00?000?110**	**?1???02001**	**0011011001**	**0101110100**	**0010000000**
Trigonias osborni	**200101?110**	**?110102101**	**0011011001**	**0101110101**	**0110000003**
Subhyracodon occidentalis	**201101?110**	**?110102111**	**1?11011001**	**0111111???**	**1110000003**

Taxa	11111 11111<br>0000000001<br>1234567890	11111 11111<br>11111 11112<br>1234567890	11111 11111<br>2222222223<br>1234567890	11111 11111<br>3333333334<br>1234567890	11111 11111<br>4444444445<br>1234567890
Heptodon posticus	0????0???0	0000???000	0000000000	0?00?00001	0010000?00
Hyrachyus eximius	0000000000	0000001001	0000100000	0?00?00101	0100001000
Hyrachyus princepts	0000011110	0000000000	0000100100	0-00-001??	??????????
Rostriamynodon grangeri	00000?11?1	10000?????	0000112?11	0?10?111??	??????????
Amynodon_advenus	0000001101	1000000000	0000112?11	0?10?111??	??????????
Forstercooperia totadentata	1000011111	1110100000	01?2100001	1?11?001??	??????????
Pappaceras confluens	1000011111	1110100100	00?1100001	0?10?001??	??????????
Pappaceras minuta	1000011111	1110100100	0010101001	0?10?001??	??????????
Papaceras meiomenus	1000001111	1110100000	0010??????	??????????	??????????
Triplopus cubitalis	0110000201	1101000000	0010100000	0?00?00101	0?01???111
Hyracodon nebraskensis	2313100211	1111110111	0011102?01	21121001?1	0101111111
Prohyracodon meridionale	0111100201	1111000000	11?2?????1	??1??0?1??	??????????
Epitriplopus uintensis	2313100201	1111100100	11?2100001	21121001??	??????????
Triplopides rieli	2313100201	1111?0?100	11?2102?01	21121001??	??????????
Ardynia praecox	2313100201	1111?0?100	11?2102?01	21121201??	??????????
Eggysodon osborni	0100000201	100000?000	01?2101010	21021201??	??????????
Allacerops turgaica	0100000201	100000?000	01?2101010	21021201??	??0???????
Proeggysodon qiui	??????????	??????????	????101000	0?00?001??	??????????
Juxia sharamurenensis	1100001111	1000100000	01?2100011	1011?00100	1001101120
Urtinotherium intermedium	2??00??1?1	10000?????	????100011	21121001??	??????????
Paraceratherium grangeri	2100000111	1000000000	01?2102011	21121001??	??0???????
Uintaceras radinskyi	0000010111	1100100000	0010100001	0?10?001??	??011111??
Teletaceras borissiaki	1000000001	1010000000	01?2100011	10110001??	??????????
Trigonias osborni	2313100211	1010000000	01?2100011	1112100110	1001101100
Subhyracodon occidentalis	2313100211	1010100000	01?2102011	1112100110	1001101100

Taxa	11111 11111<br>5555555556<br>1234567890	11111 11111<br>6666666667<br>1234567890	11111 11111<br>7777777778<br>1234567890	11111 11111<br>8888888889<br>1234567890	111111111 2222<br>9999999990000<br>1234567890123
Heptodon posticus	000?0000?0	0100010000	0000000100	0?000??000	00000010???00
Hyrachyus eximius	0100000000	0?1??01020	0000000100	0100000000	0?00000000000
Hyrachyus princepts	??????????	??????????	??????????	??????????	?????????????
Rostriamynodon grangeri	??????????	??????????	??????????	??????????	???1?????????
Amynodon_advenus	???????1??	??????????	?????0????	??????????	???1?????????
Forstercooperia totadentata	??????????	??????????	??????????	??????????	?????????????
Pappaceras confluens	??????????	??????????	??????????	??????????	?????????????
Pappaceras minuta	??????????	??????????	??????????	??????????	?????????????
Papaceras meiomenus	??????????	??????????	??????????	??????????	?????????????
Triplopus cubitalis	1101?10010	1???11?000	?00101??11	??????????	???????????11
Hyracodon nebraskensis	1101?10010	1??0?1?000	000101?111	1000100000	0000?1?101111
Prohyracodon meridionale	??????????	??????????	??????????	??????????	?????????????
Epitriplopus uintensis	??????????	??????????	??????????	??????????	???????????11
Triplopides rieli	??????????	??????0???	??????????	??????????	???????????11
Ardynia praecox	??????????	??????0???	??????????	??????????	???????????11
Eggysodon osborni	??????????	00?10001??	??????????	?????????0	1101????00??1
Allacerops turgaica	???0010???	01?10001??	???01?????	10100????0	1??1011????21
Proeggysodon qiui	??????????	??????????	??????????	??????????	?????????????
Juxia sharamurenensis	000?000111	0000110101	0????10100	0010010110	1??1?11101100
Urtinotherium intermedium	??????????	??????????	??????????	?????????0	0?1????????2?
Paraceratherium grangeri	??????????	??????????	??1??21??1	121110?1?1	0?11???????21
Uintaceras radinskyi	??1011001?	0?01?00??0	1100021000	?1?011001?	00?1100????20
Teletaceras borissiaki	??????????	??????????	?????0?011	??????????	?10?101????21
Trigonias osborni	0010111110	0101010110	1100021011	01100??111	1101100?10021
Subhyracodon occidentalis	0010111110	0101010110	1100021011	0110001111	?101100?00121

3. Synapomorphies

Table 1 Distribution of the synapomorphies in the most-parsimonious tree

Node	Characters and state
27	42(1), 56(1)
28 (A)	40(1), 43(1), 49(1), 66(1), 96(2), 98(1), 99(1), 126(1), 127(1), 136(1), 137(1)
29 (J)	7(1), 22(1), 24(1), 32(1), 39(1), 44(1), 47(1), 72(1), 95 (1)
30	9(1), 35(1), 36(1), 70(1), 88(2), 140(0), 141(1), 158(1), 168(1), 172(1), 183(1), 189(1), 194(1)
31	76(1), 110(1), 111(1)
32	106(1)
33 (F)	1(0), 113(1)
34 (P)	30(1), 38(1), 43(2), 54(1), 64(1), 101(1)
35	1(2), 29(1), 63(1), 94(1), 109(1), 115(1), 176(2)
36	118(1)
37 (H)	102(1), 103(1), 112(1), 149(1), 150(1), 151(1), 154(1), 161(1), 174(1), 175(1)
38	127(2)
39	93(1), 100(1), 101(1), 102(1), 115(1), 117(1)
40	92(1), 105(1), 113(1), 121(1), 122(1), 124(2)
41	26(1), 96(2)
42	131(2), 136(2)
43 (E)	51(1), 52(1), 78(1), 127(1)
44 (G)	93(1), 122(1), 124(2), 191(1), 192(1)
45 (U)	4(1), 5(1), 8(1), 14(1), 29(2), 30(2), 31(1), 37(1), 73(1), 74(1), 80(1), 84(1), 85(1), 86(1), 88(1), 93(1), 100(2), 102(1), 122(1), 124(2)
46	51(2), 53(1), 68(1), 78(2), 92(1), 101(2), 114(1), 115(0)
47 (R)	62(1), 67(2), 74(1), 77(1), 80(1), 82(1), 84(1), 85(1), 86(1), 88(1), 113(1), 131(1)
48	1(2), 13(1), 19(1), 51(2), 68(1), 100(3), 102(3), 103(1), 104(3), 105(1), 109(1), 176(2), 197(0)

4. Measurements

Table 2 Measurements of cranium (IVPP V20254) (mm)

Skull and upper teeth	IVPP V20254
Basilar length	363
Profile length (total length)	*390
Distance between occipital and postorbital process	182
Distance between premaxilla and postorbital process	234
Distance between P2 and condyle	316
Distance between postglenoid process and condyle	87.6
Length of nasal notch	47.5
Distance between anterior end of orbital and nasal notch	112
Distance between premaxilla and anterior end of orbital	83.5
Greatest length of nasal	85.5
Bizygomatic breadth	182
Palatine breadth in front of P2	*34.5
Palatine breadth in front of M1	*39.8
Palatine breadth in front of M3	*43.6
Greatest breadth of postglenoid processes	115
Greatest breadth of parocciptal processes	97.3
Height of occipital	*86.8
Greatest breadth of foramen magnum	26.7
Greatest breadth of occipital condyles	*62.9

Note: estimated values are marked with asterisk (*).

Table 3 Measurements of upper teeth (IVPP V20254) (mm)

	V 20254	
	Left	Right
I1 L	7.82	
I1 W	7.50	
I2 L	7.88	7.81
I2 W	7.91	7.93
I3 L	8.50	8.48
I3 W	7.08	7.06
C L	11.8	11.8
C W	9.70	9.78
P2 L	12.7	12.5
P2 W	12.8	12.7
P3 L	13.8	13.8
P3 W	18.1	18.2
P4 L	15.8	15.6
P4 W	24.3	23.4
M1 L	24.3	24.3
M1 W	*27.0	26.4
M2 L	30.1	30.2
M2 W	27.3	27.4
M3 L	28.3	28.5
M3 W	28.0	28.2
P2-4 L	43.4	43.5
M1-3 L	77.6	80.9
P1-M3 L	120.6	123.9
Diastema between C and P2	24.1	30.2

Note: L is for length, and W for width. Estimated values are marked with asterisk (*).

5. References

1 Antoine, P. O. *et al.* A revision of Aceratherium blanfordi Lydekker, 1884 (Mammalia: Rhinocerotidae) from the Early Miocene of Pakistan: postcranials as a key. *Zool J Linn Soc-Lond* **160**, 139-194, doi:10.1111/j.1096-3642.2009.00597.x (2010).

2 Holbrook, L. T. The phylogeny and classification of Tapiromorph Perissodactyls (Mammalia). *Cladistics* **15**, 331-350, doi:DOI 10.1006/clad.1999.0107 (1999).

3 Qiu, Z. X. & Wang, B. Y. *Paracerathere fossils of China*. 1-387 (Science Press, 2007).

4 Holbrook, L. T. & Lucas, S. G. A new genus of Rhinocerotoid from the Eocene of Utah and the status of North American "Forstercooperia". *J Vertebr Paleontol* **17**, 384-396 (1997).
